# Association between dietary folate intake and the risk of osteoporosis in adults: a cross-sectional study

**DOI:** 10.1186/s12891-024-07605-9

**Published:** 2024-06-22

**Authors:** Li Zhou, Weinmin Deng, Qingrong Wu, Yandong Pan, Hongxing Huang

**Affiliations:** 1 The Third School of Clinical Medicine, Guangzhou University of Chinese Medicine, Guangzhou, Guangdong 510405 China; 2 Department of Rehabilitation and Physiotherapy, Foresea Life Insurance, Guangzhou General Hospital, Guangzhou, Guangdong 510000 China; 3 Department of Rehabilitation and Physiotherapy, General Hospital of the Southern Theater of the Chinese People’s Liberation Army, Guangzhou, Guangdong 510000 China; 4 Department of Pharmacy, Ganzhou Fifth People’s Hospital, Ganzhou, Jiangxi 341000 China; 5 Department of Integrated Traditional Chinese and Western Medicine, Guangzhou Dongsheng Hospital, Guangzhou, Guangdong 510000 China; 6 The Third Affiliated Hospital of Guangzhou University of Chinese Medicine, Guangzhou, Guangdong 510375 China

**Keywords:** Dietary folate intake, Osteoporosis, Nonlinear relationship, Cross-sectional study

## Abstract

**Background:**

Increased intake of specific vitamins has been linked to a decreased prevalence of osteoporosis. However, the association between dietary folate intake and the risk of osteoporosis in the general population remains incompletely understood. Therefore, we aimed to determine the association between dietary folate intake and the risk of osteoporosis in the general population of the USA.

**Methods:**

In this cross-sectional study, data from the National Health and Nutrition Examination Survey (2017–2020) were collected. Osteoporosis was considered to be indicated by a bone mineral density greater than 2.5 standard deviations below the mean of the young adult reference group. Dietary folate intake was measured by a 24-hour dietary recall. Multivariate logistic regression models and restricted cubic spline models were used.

**Results:**

The study included 2297 participants (mean age: 63.69 ± 0.35 years), 49.92% of whom were female. In the general population, increased dietary folate intake was directly associated with a decreased risk of osteoporosis (*P* for trend = 0.005). In the age > 60 years and female subgroups, folate intake was inversely associated with the risk of osteoporosis (*P* for trend < 0.001). The dose‒response curve suggested that this association was nonlinear (*P* for nonlinearity = 0.015).

**Conclusions:**

Our cross-sectional study provides initial insights into the inverse association between dietary folate intake and the risk of osteoporosis in the general U.S. population. Further research is needed to confirm these associations.

## Introduction

Osteoporosis, a systemic skeletal disorder, is characterized by reduced bone mineral density (BMD) and changes in bone microstructure. This disease leads to increased bone fragility, significantly increasing the risk of severe fractures and disability. With the aging of the global population, osteoporosis is becoming more common among older adults and has become a prominent contributor to fracture-related morbidity and mortality [1]. Osteoporosis patients experience an average of 5.8 disability-adjusted life years throughout disease progression [2]. Fractures due to osteoporosis in older adults often require extended care and impose substantial medical and economic burdens, posing a formidable global public health challenge [3, 4].

Life patterns and nutrients are widely acknowledged to significantly influence bone health [5–7]. Calcium deficiency is an important cause of osteoporosis. In the anti-osteoporosis guidelines, calcium and vitamin D supplements are recommended as key anti-osteoporosis nutrients for maintaining bone health as well as preventing and treating osteoporosis [8]. Exercise also plays an important role in improving osteoporosis. Jing Liu, et al. reported that calcium and vitamin D supplementation-based regular sling core stabilization training improved bone density in osteoporosis patients [9]. In addition, the elderly suffer from osteoporosis. A systematic review of 201 older adults, including six studies, revealed that dietary supplements had a positive impact on mitochondrial oxidative and antioxidant capacity, volume, bioenergy capacity, and mitochondrial transcriptome [10]. This helps mitochondria regulate the balance between osteogenic and osteoclastic activity, maintain bone homeostasis, which may reduce osteoporosis in the elderly population [11]. A recent study conducted in the elderly population indicates that obesity is a significant risk factor for knee osteoarthritis, and weight reduction achieved through the use of dietary supplements, dietary control strategies, and exercise can effectively alleviate knee osteoarthritis [12]. Those studies demonstrated the beneficial impact of dietary supplements on bone health in the elderly population.

Folate is a common dietary supplement, also known as vitamin B9, which is indispensable for bone health. A previous study revealed that elevated serum folate levels are linked to increased BMD [13]. Folate influences the metabolism of homocysteine, an amino acid implicated in bone integrity, both directly and indirectly [14]. Studies have indicated that osteoporosis is significantly related to the level of circulating homocysteine in the body [15, 16]. High levels of homocysteine can enhance osteoclast activity and differentiation [17] and induce apoptosis in human bone marrow stromal cells produced by reactive oxygen species. It can also interrupt collagen crosslinking development and reduce bone blood flow, affecting bone tissue formation [18, 19] and ultimately leading to poor iliac health and brittle fractures. The level of homocysteine is affected by various B vitamins, including folate. Folate deficiency leads to an increase in homocysteine levels, thereby reducing BMD and increasing osteoporosis risk [20]. Consequently, augmenting dietary intake of folate may be a crucial strategy for preventing osteoporosis. However, few observational studies have been conducted to investigate the association between folate intake and osteoporosis risk. To our knowledge, one cross-sectional study revealed a significant positive association between dietary folate intake and BMD [21]. To date, the association between folate intake and the risk of osteoporosis has not been assessed extensively among adults in the general population.

In this study, we investigated the relationship between dietary folate intake and osteoporosis risk using cross-sectional data from the National Health and Nutritional Examination Survey (NHANES).

## Methods

### Study design

This study employed a cross-sectional design, and the study data were derived from the NHANES, a cross-sectional, stratified, multistage program survey. The aim of the NHANES is to evaluate the health and nutritional status of American adults and children. In brief, approximately 5000 participants are recruited each year to form a nationally representative sample from 15 counties across the country through multistage probability sampling. Each participant completed a uniformly structured questionnaire and examination. The National Center for Health Statistics Ethics Review Board approved the NHANES, and all participants provided informed consent. In this study, we used the NHANES 2017–2020 data.

### Study population

In this study, the inclusion criteria were individuals aged eighteen years and above in the NHANES 2017–2020. The exclusion criteria were participants with missing data on DXA absorptiometry of the femur, dietary folate intake, and covariates of interest (including age, sex, race, education, marital status, poverty income ratio, hypertension, diabetes, dietary vitamin D intake, dietary calcium intake, and dietary phosphorus intake). To ensure an enough sample size, no further exclusions were made.

### Definition of osteoporosis

The outcome of this study was osteoporosis. The BMD of the total femur, femoral neck, trochanter, and intertrochanter was assessed by DXA, which was administered by trained and certified radiologists. A Hologic Discovery Model A densitometer (Hologic, Inc., Bedford, Massachusetts) was used to obtain the scans. We routinely scanned the left hip unless the participant reported a fracture, hip replacement, or a pin in the left hip. Participants who were pregnant, had a self-reported history of radiographic contrast material, or whose weight exceeded 204.12 kg were excluded from the DXA examination.

According to a previous study [22], osteoporosis was characterized by BMD values that fell more than 2.5 standard deviations lower than the average of the reference group, which consisted of individuals aged 20–29 years from the NHANES III dataset. In this study, osteoporosis was evaluated in four regions of the femur, namely, the total femur, trochanter, intertrochanter, and femur neck.

In this study, osteoporosis was defined as a BMD < 0.64 g/cm^2^ for women and < 0.68 g/cm^2^ for men in the total femur region, < 0.56 g/cm^2^ for women and < 0.59 g/cm^2^ for men in the femur neck, < 0.46 g/cm^2^ for women and < 0.49 g/cm^2^ for men in the trochanter, < 0.74 g/cm^2^ for women and < 0.78 g/cm^2^ for men in the intertrochanter. Total osteoporosis is defined as osteoporosis occurring in any region of the femur.

### Dietary folate intake

The independent variable in this study was dietary folate intake. The dietary interviewer measured dietary folate intake and other nutrients via 24-hour recall interviews, and the use of a respondent-driven approach allowed us to collect accurate and detailed information on the types and quantities of foods and beverages consumed within the 24-hour period prior to the interview. The 24-hour dietary recalls were conducted twice. The first recall was performed face to face at the Mobile Examination Center. After 3 to 10 days, the second recall was performed via telephone. The Argenfoods nutrient composition database and the United States Department of Agriculture database were used to determine the nutrient composition of the recalled food items. The Automated Multiple-Pass Method was utilized to compute the nutrient intake data. The average folate intake from these two recall interviews was used and assessed in mcg per day (mcg/day).

### Assessment of covariates

We considered the following potential confounders: sociodemographic variables (including age, sex, race, education, marital status, and poverty income ratio), disease status (including hypertension and diabetes) and dietary intake factors (including vitamin D, calcium, and phosphorus). Sociodemographic variables and disease information were obtained from questionnaires administered by trained interviewers using a computer-assisted personal interview (CAPI) system.

The poverty income ratio was defined as ≥ 1 for individuals categorized as not poor and < 1 for individuals categorized as poor [23]. Smoking, alcohol consumption, hypertension, and diabetes data were obtained from the questionnaire. Smoking status was assessed as nonsmoker or smoker. Nonsmokers were defined as those who smoked fewer than 100 cigarettes in their life, while others were defined as smokers [24]. Alcohol consumption was categorized as never (never drinking in the last year), occasional (drinking fewer than once a week), or frequent (drinking at least once a week) [25]. Hypertension status and diabetes status were dichotomized as yes or no. Dietary intakes of vitamin D, calcium, and phosphorus were obtained from dietary interview components using methods similar to those described above for dietary folate intake.

### Statistical analysis

Sampling weights were used in all analyses, following the suggestion to account for the complex survey design of the NHANES. Continuous variables are presented as the mean ± standard error, and categorical variables are presented as counts (weighted percentages). Differences in characteristics between the two groups of participants with or without osteoporosis were evaluated using Student’s *t* test for continuous variables and chi-square tests for categorical variables.

The dietary folate intake levels were categorized into tertiles: lowest (≤ 264 mcg/day), middle (264–390 mcg/day), and highest (> 390 mcg/day). The lowest group of dietary folate intake served as the reference group. Logistic regression models were used to calculate the odds ratio (OR) with 95% confidence interval (Cl) for the association of dietary folate intake levels with the risk of osteoporosis. The logistic models were first adjusted for age, sex, race, education, marital status, and poverty income ratio and then adjusted for other covariates, including smoking status, alcohol consumption status, hypertension status, diabetes status, dietary vitamin D intake, dietary calcium intake, and dietary phosphorus intake. To assess the dose‒response associations between dietary folate intake and the risk of osteoporosis, we used restricted cubic spline models with 3 knots placed at the 5th, 50th, and 95th percentiles as recommended. Because of the sparsity of the data, we truncated the analysis to 750 mcg/day (the outlier upper bound). Subgroup analyses stratified by sex and age (< 60 and ≥ 60 years) in terms of the relationship between dietary folate intake and the risk of osteoporosis were also conducted.

All analyses were performed with Stata SE, version 15.0 (Stata Corp LP., College Station, Texas, USA), along with the Storm Statistical Platform (www.medsta.cn/software). Two-tailed *P* values less than 0.05 were considered to indicate statistical significance.

## Results

**Fig. 1:**
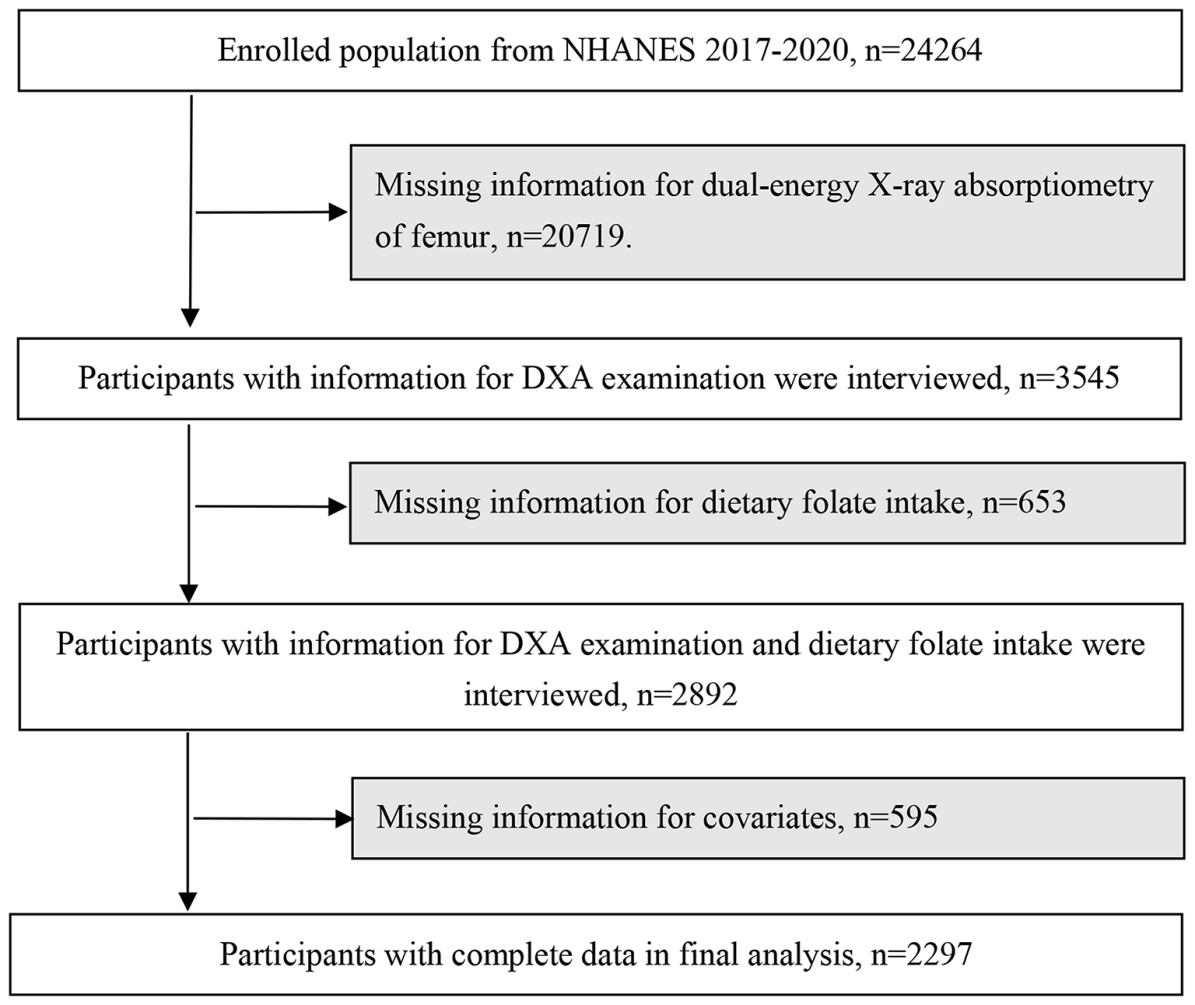
Flowchart of the study participants

A total of 24,264 participants were recruited, and we excluded 2567 participants whose data for DXA femur (*n* = 20,719) or dietary folate intake (*n* = 653) were missing. We further excluded 595 individuals with missing information for covariates of interest. Finally, 2297 participants were available for analysis in this study. The flowchart of the study participants is shown in Fig. 1.

### Characteristics of the study population

Among the 2297 participants included in this cross-sectional study, 49.92% were female, with a mean age of 63.69 years. Additionally, 159 participants met the criteria for osteoporosis diagnosis. The overall weighted prevalence of osteoporosis was found to be 6.92%. Compared with individuals without osteoporosis, those with osteoporosis were more likely to be older, female, Non-Hispanic White, and widowed/divorced/separated and have nondrinker status and lower dietary folate intake. The differences in education, family income, smoking status, dietary vitamin D/calcium/phosphorus intake, hypertension status, and diabetes status between the two groups were not significant (Table 1).

**Table 1:** Characteristics of study participants

Characteristics	Total (*N* = 2297)	Without osteoporosis (*N* = 2138)	With osteoporosis (*N* = 159)	*P*
Age (years)	63.69 ± 0.35	63.14 ± 0.34	70.15 ± 0.50	< 0.001
Female	1053 (49.92)	943 (47.67)	110 (76.47)	< 0.001
Race/Ethnicity				< 0.001
Mexican American	196 (4.26)	191 (4.52)	5 (1.22)	
Other Hispanic	243 (6.24)	237 (6.59)	6 (2.22)	
Non-Hispanic White	999 (73.33)	884 (72.37)	115 (84.69)	
Non-Hispanic Black	613 (9.16)	595 (9.64)	18 (3.63)	
Other race	246 (6.99)	231 (6.89)	15 (8.24)	
Education level				0.136
Less than 9th grade	125 (2.26)	118 (2.34)	7 (1.34)	
9-11th grade	216 (6.21)	205 (6.24)	11 (5.82)	
High school	580 (30.05)	522 (29.25)	58 (39.45)	
Some college	777 (29.62)	733 (30.35)	44 (21.04)	
College graduate	599 (31.86)	560 (31.82)	39 (32.35)	
Marital status				0.009
Married/Living with Partner	1373 (66.32)	1289 (67.47)	84 (52.77)	
Widowed/Divorced/Separated	726 (27.38)	664 (26.22)	62 (41.04)	
Never married	198 (6.30)	185 (6.31)	13 (6.19)	
Poverty income ratio				0.736
Poor	335 (8.71)	315 (8.67)	20 (92.24)	
Not poor	1962 (91.29)	1823 (91.33)	139 (90.76)	
Smoking status				0.563
Yes	1161 (49.48)	1075 (49.25)	86 (52.26)	
No	1136 (50.52)	1063 (50.75)	73 (47.74)	
Alcohol consumption				0.019
Never	654 (22.32)	588 (21.16)	66 (36.03)	
Occasional	935 (42.40)	882 (42.86)	53 (36.90)	
Frequent	708 (35.28)	668 (35.98)	40 (27.07)	
Folate (continuous) (mcg/day)	354.68 ± 4.87	359.61 ± 5.02	296.40 ± 13.48	< 0.001
Folate (tertiles)				< 0.001
Lowest	764 (31.73)	693 (30.16)	71 (50.12)	
Middle	766 (33.84)	716 (33.91)	50 (33.05)	
Highest	767 (34.43)	729 (35.93)	38 (16.83)	
Dietary Vitamin D intake (mcg)	4.56 ± 0.16	4.50 ± 0.13	5.29 ± 1.16	0.497
Dietary calcium intake (mg)	906.75 ± 16.41	910.53 ± 17.07	862.14 ± 56.25	0.411
Dietary phosphorus intake (mg)	1327.95 ± 19.40	1338.77 ± 19.52	1200.14 ± 77.55	0.086
Hypertension				0.363
Yes	1245 (49.50)	1164 (49.89)	81 (44.96)	
No	1052 (50.50)	974 (50.11)	78 (55.04)	
Diabetes				0.393
Yes	1758 (80.53)	1627 (80.20)	131 (84.39)	
No	539 (19.47)	511 (19.80)	28 (15.61)	

Continuous variables are presented as the means ± standard errors (SEs); categorical variables are presented as counts (weighted percentages)

### Association between dietary folate intake and the risk of osteoporosis

Table 2 shows the weighted ORs and trends for the association between dietary folate intake and the risk of osteoporosis after logistic regression modeling. According to Model 1, compared with those in the lowest tertile of dietary folate intake, the ORs (95% CIs) of incident osteoporosis were 0.70 (0.38, 1.27) and 0.39 (0.22, 0.69) (*P* for trend = 0.005), respectively. Similar results were found in Model 2 when every covariate of interest was adjusted.

**Table 2:** Weighted odds ratios (ORs) and 95% confidence intervals (CIs) for the association between dietary folate intake and the risk of osteoporosis

Dietary folate intake (tertiles)	Total	Cases (%)		Model 1^a^			Model 2^b^	
				OR (95% CI)^a^	*P* value		OR (95% CI)^b^	*P* value
Lowest	764	71 (9.29%)		Reference			Reference	
Middle	766	50 (6.53%)		0.70 (0.38, 1.27)	0.228		0.64 (0.34, 1.19)	0.154
Highest	767	38 (4.95%)		0.39 (0.22, 0.69)	0.002		0.30 (0.15, 0.58)	0.001
*P* for trend	2297	159 (6.92%)			0.005			0.002

^a^ Adjusted for age, sex, race, education, marital status, and poverty income ratio

^b^ Adjusted for age, sex, race, education, marital status, poverty income ratio, smoking status, alcohol consumption status, hypertension status, diabetes status, dietary vitamin D intake, dietary calcium intake, and dietary phosphorus intake

### Dose–response relationship between dietary folate intake and the risk of osteoporosis

**Fig. 2:**
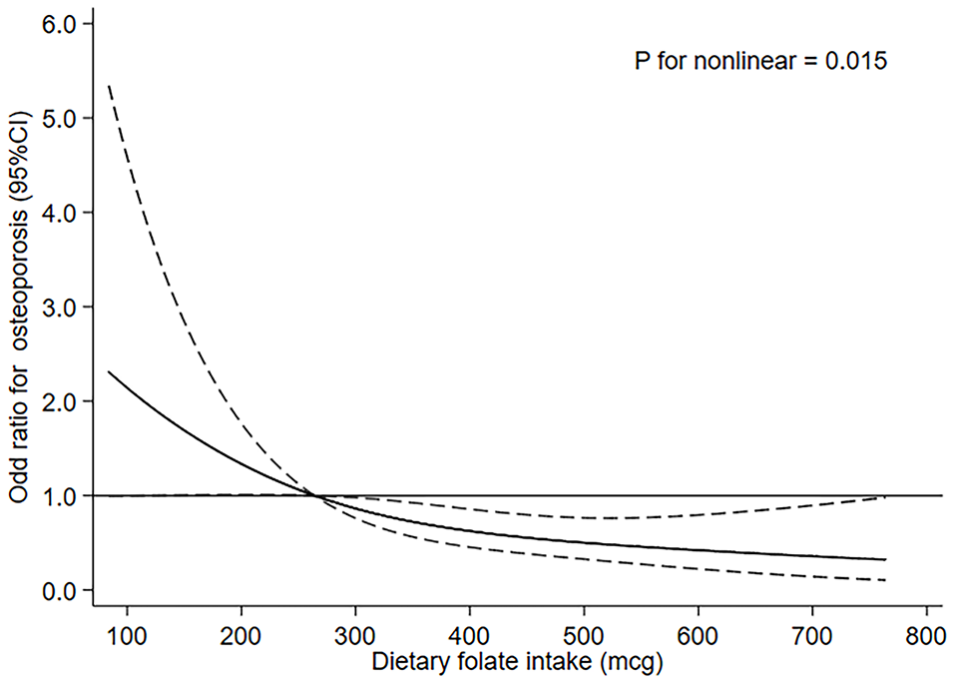
The dose‒response relationship between dietary folate intake and the risk of osteoporosis

Figure 2 shows the results of the restricted cubic spline analyses and suggests an L-shaped relationship between dietary folate intake and the risk of osteoporosis. The risk of osteoporosis decreased with increasing dietary folate intake and showed nonlinear dose–response associations (*P* for nonlinearity = 0.015).

### Relationship between dietary folate intake and the risk of osteoporosis according to sex and age in the subgroup analysis

Table 3 shows the results of the subgroup analysis stratified by sex and age. The results demonstrated that dietary folate intake was strongly related to the risk of osteoporosis among females (OR = 0.18, 95% CI: 0.06–0.60, *P* = 0.007) and individuals aged ≥ 60 years (OR = 0.30, 95% CI: 0.16–0.54, *P* < 0.001). However, the associations of dietary folate intake with the risk of osteoporosis were negative among males (OR = 0.60, 95% CI: 0.23–1.57, *P* = 0.286) and participants aged < 60 years (OR = 0.55, 95% CI: 0.04–7.24, *P* = 0.640).

**Table 3:** Weighted odds ratios (ORs) and 95% confidence intervals (CIs) for the association between dietary folate intake and the risk of osteoporosis by age and sex

	Dietary folate intake (mcg)	Total	Cases (%)		OR (95% CI) ^a^	*P* value
Male	Lowest	311	15 (4.82%)		Reference	
	Middle	427	12 (2.81%)		0.70 (0.20, 2.53)	0.576
	Highest	506	22 (4.35%)		0.60 (0.23, 1.57)	0.286
	*P* for trend	1244	49 (3.94%)			0.270
Female	Lowest	453	56 (12.36%)		Reference	
	Middle	339	38 (11.21%)		0.63 (0.32, 1.25)	0.178
	Highest	261	16 (6.13%)		0.18 (0.06, 0.60)	0.007
	*P* for trend	1053	110 (10.45%)			0.007
Age < 60	Lowest	252	12 (4.76%)		Reference	
	Middle	236	5 (2.12%)		0.57 (0.11, 2.81)	0.474
	Highest	276	7 (2.54%)		0.55 (0.04, 7.24)	0.640
	*P* for trend	764	24 (3.14%)			0.630
Age ≥ 60	Lowest	512	59 (11.52%)		Reference	
	Middle	530	45 (8.49%)		0.65 (0.35, 1.19)	0.158
	Highest	491	31 (6.31%)		0.30 (0.16, 0.54)	< 0.001
	*P* for trend	1533	135 (8.81%)			< 0.001

^a^ Adjusted for age, sex, race, education, marital status, poverty income ratio, smoking status, alcohol consumption status, hypertension status, diabetes status, dietary vitamin D intake, dietary calcium intake, and dietary phosphorus intake

## Discussion

This study investigated the relationship between dietary folate intake and the risk of osteoporosis. The study revealed a nonlinear negative dose‒response relationship, suggesting that the effect of dietary folate on osteoporosis risk changes in magnitude across different levels of intake. Moreover, the subgroup analysis showed that significant associations between dietary folate intake and the risk of osteoporosis were primarily found in women or participants aged ≥ 60 years. These findings suggest that increased dietary folate intake might be linked to a diminished risk of developing osteoporosis, especially among older individuals and females.

Folate is a water-soluble vitamin that prevents DNA damage, reduces oxidative stress and apoptosis, and has multiple benefits for various physiological systems. It is critical in skeletal diseases [26, 27]. Studies have shown a significant positive correlation between folate intake and BMD [21], and folate is also associated with serum homocysteine levels; both decreased BMD and increased homocysteine are risk factors for osteoporosis and osteoporotic fractures. Folate is a key coenzyme that degrades homocysteine through the remethylation and sulfur transfer pathways. Consequently, folate deficiency can lead to a surge in homocysteine levels [28]. Elevated levels of plasma homocysteine have been demonstrated to impact collagen crosslinking, thereby impairing bone strength [29]. In addition, an experimental study showed increased osteoclast count and activity in cells supplemented with folate compared to cells cultured without folate [30]. Another animal experimental study in mice also revealed that the activity of osteoclasts increased in mice supplemented with folate, along with an increase in fat cells. Abnormalities in lipid and glucose metabolism are closely linked to osteoporosis, suggesting that folate may influence osteoporosis occurrence through its regulation of lipid metabolism [31]. These findings suggest that folate has a direct role in protecting bone health.

In this study, we found that high dietary folate intake was associated with increased osteoporosis risk, and this association was more significant in women and older individuals. There are various explanations for this phenomenon. Studies have shown that bone resorption peaks five to ten years after the onset of menopause, compared to the years before and after menopause [32]. On the other hand, the level of homocysteine, an important risk factor for bone health, increases with age [14, 33] and is greater in postmenopausal women than in premenopausal women [34]. Homocysteine is inversely associated with folate and vitamin B12 levels, suggesting that folate contributes significantly to the onset of osteoporosis in older women [14]. A study of older white, black, and Mexican-American women in the United States (> 50 years of age) revealed a positive linear association between folate content and overall BMD [35]. These studies suggest that folate is a significant factor affecting the bone health of older women and that increased dietary folate may be effective in preventing osteoporosis in women. However, the correlation is not always consistent. A study of young postmenopausal Turkish women revealed no correlation between lumbar BMD and folate or vitamin B-12 intake, with no difference in the plasma folate concentration between women with normal BMD and those with osteoporosis and osteopenia [32]. Overall, these studies offer some valuable insights, but the impact of folate on bone health remains uncertain, and the effectiveness of dietary folate supplementation in preventing osteoporosis in women needs to be confirmed by further homogeneous studies.

The dose‒response relationship showed that increasing dietary folate intake was associated with a reduced risk of osteoporosis in this study. However, it has been reported that the recommended dietary intake of folate is 400 µg/d, and the tolerable upper intake is 1000 µg/d [36]. Recent research has identified the risk of excess folate in the context of vitamin B12 deficiency in the nervous system, especially regarding cognitive function [37]. Another study on the connection between folate and cancer revealed that folate plays a dual regulatory role in carcinogenesis [38]. In individuals with folate deficiency or in the early stage of cancer, appropriate folate intake can prevent tumor development; however, in individuals with high levels of folate intake or in a precancerous state, folate promotes tumor development. The results of this study showed that folate had a protective effect against osteoporosis in the participant population when the daily dietary intake of folate was over 264 µg/d (OR < 1). Although this study was not able to show an upper limit for folate intake in the prevention of osteoporosis, another study suggested that postmenopausal women should not consume more than 528 ~ 569 µg of dietary folate daily for optimal bone health [21]. Therefore, dietary folate intake ranging from 264 to 569 µg/day may be effective in preventing osteoporosis in postmenopausal women, which is similar to the level of dietary folate recommended by the National Academy of Sciences, but further exploration is needed.

This study has several limitations. First, as a cross-sectional analysis, our results cannot be used to establish a causal relationship between dietary folate intake and the onset of osteoporosis. Further longitudinal studies are needed to explore the potential causal relationship between dietary folate intake and the risk of osteoporosis. Second, the research data were primarily from self-report questionnaires and interviews, which may introduce recall bias. However, in the NHANES, recall bias is minimized by conducting interviews and examinations in a standardized manner by trained professionals. Especially in dietary recall, all data collection is divided into five steps that are used to collect various information about foods consumed during the 24-hour period of the previous day. The various steps prompt respondents to consider their intake in different ways and from different perspectives, with the aim of maximizing respondents’ likelihood of recalling and reporting foods they have consumed. Additionally, we only used dietary data from two complete 24-hour recall interviews to further reduce recall bias. While several potential confounding variables were adjusted for, our results may also be influenced by other unmeasured potential confounding variables. Thus, further studies need to take these factors into account to validate our findings.

## Conclusion

In summary, this study provides valuable insight into the cross-sectional association between lower dietary folate intake and increased osteoporosis risk in the general American population. This finding suggests the potential importance of dietary folate intake for preventing and managing osteoporosis. However, further longitudinal research and randomized controlled trials are necessary to elucidate the causal association between dietary folate intake and the risk of osteoporosis. Additionally, validation of these findings across diverse populations and settings is warranted.

## Data Availability

The datasets used and/or analyzed during the current study are available from the corresponding author on reasonable request.

## Abbreviations

BMD: Bone Mineral Density
NHANES: National Health and Nutritional Examination Survey
DXA: Dual-energy X-ray Absorptiometry
OR: Odds Ratio
CI: Confidence Interval
